# The last phase of life with dementia in Swiss nursing homes: the study protocol of the longitudinal and prospective ZULIDAD study

**DOI:** 10.1186/s12904-016-0151-2

**Published:** 2016-08-24

**Authors:** Stefanie Eicher, Nathan Theill, Heike Geschwindner, Caroline Moor, Albert Wettstein, Gabriela Bieri-Brüning, Christoph Hock, Mike Martin, Henrike Wolf, Florian Riese

**Affiliations:** 1 University Research Priority Program “Dynamics of Healthy Aging”, University of Zurich, Andreasstrasse 15, 8050 Zurich, Switzerland; 2 Center for Gerontology, University of Zurich, Pestalozzistrasse 24, 8032 Zurich, Switzerland; 3 Division of Psychiatry Research and Psychogeriatric Medicine, University of Zurich, Lenggstr. 31, 8032 Zurich, Switzerland; 4 City of Zurich Nursing Homes, Walchestrasse 31, 8021 Zurich, Switzerland; 5 Municipal Physician Service Zurich, Walchestrasse 31, 8021 Zurich, Switzerland; 6 Department of Psychology, University of Zurich, Binzmuehlestrasse 14, 8050 Zurich, Switzerland

**Keywords:** Advanced dementia, Palliative care, End-of-life care, Nursing home, Satisfaction with care, Quality of care, Qquality of life, Dying, Terminal phase

## Abstract

**Background:**

The proportion of older people with advanced dementia who will die in nursing homes is constantly growing. However, little is known about the dying phase, the type of symptoms, the management of symptoms and the quality of life and dying in people with advanced dementia. The ZULIDAD (Zurich Life and Death with Advanced Dementia) study aims at extending the current scientific knowledge by providing first data from Switzerland.

**Methods:**

The ZULIDAD study employs a prospective design to study nursing home residents with advanced dementia for three years or until their death in eleven nursing homes in Zurich. Observational data from quarterly questionnaires for relatives and primary nurses is combined with data from the Resident Assessment Instrument – Minimum Data Set (RAI-MDS). Special focus is put on 1) the cross-sectional analysis of baseline and post-mortem data regarding quality of life and quality of dying and how the perceptions of these measures differ between relatives and primary nurses, 2) the longitudinal analyses of established health outcome measures (e.g., EOLD, MSSE, BISAD, QUALID) in order to understand their trajectories and 3) international comparisons of cross-sectional and longitudinal data.

**Discussion:**

The ZULIDAD study is one of the few existing prospective studies on end-of-life care in dementia and it is the first prospective study to describe the situation in Switzerland. Its multi-perspective approach allows a comprehensive approximation to central health outcome measures at the end of life such as pain, suffering or quality of life. Providing insights into the current provision of care, it can serve as a basis for improving dementia end-of-life care in Switzerland and internationally.

## Background

In the year 2010, 35 million people were estimated to live with dementia worldwide and it is predicted that this number will double every 20 years until 2050 [1]. Even though the percentages differ between countries, the majority of people with advanced dementia die in long-term care facilities (67 % in the U.S. [2]; 50.2 % in Wales, 92 % in Netherlands [3]; 30.1 % in Germany [4]). The last months of life of nursing home residents with advanced dementia (RAD) are frequently accompanied by distressing symptoms such as dyspnea, pain, pressure ulcers and eating problems, and these symptoms increase with the proximity of death [5, 6]. Due to behavioral problems, unidentified pain, inappropriate medication or other factors, quality of life can be impaired [7]. Even though there is little difference between those people dying with dementia and those dying without cognitive impairments regarding burdensome symptoms in the last phase of life [8] and causes of death [9], RAD are frequently not perceived as having a terminal condition and mostly do not receive optimal palliative care [5, 7, 10]. In order to emphasize the appropriateness and necessity of palliative care in dementia, the European Association of Palliative Care issued a white paper which attempts to define high quality palliative care in dementia [11]. However, only some of the white paper’s recommendations have been included in national dementia strategies so far [12].

The increasing amount of studies in the field motivated van der Steen and Goodman [13] to point out what kind of research is needed in order to advance in research as well as in practical care regarding dementia at the end of life. Besides the demand for theory-driven research, they advocate for study designs which allow for comparisons either with other patient groups, health care systems or countries and for analyses of disease trajectories. Furthermore they advocate for multidisciplinary research. The latter is important not only because palliative care is per se multidisciplinary, but also because RAD are unable to reliably communicate their wellbeing, discomfort or care preferences. The conflation of different proxy estimations, e.g., from relatives (REL), primary nurses (PN) or physicians regarding central outcome measures such as pain, suffering, quality of life is essential in order to provide optimal individualized care. So far, most studies regarding palliative care in dementia have been small and only few have applied a prospective design that allows the determination of disease trajectories and the concomitant proxy estimations [14] (Table 1). Furthermore, the four published prospective studies often used different outcome measures in different proxy groups and this hampers the direct comparison of proxy estimations. As a consequence, further multi-perspective prospective studies on RAD are needed, especially in Switzerland, where data is completely lacking. Exploratory research adapted to the Swiss cultural context and the local structures of the health care system can shed light on the current state of dementia palliative care in Switzerland. Furthermore, in accordance with current recommendations on research strategies in the field [13], for the present study data is prospectively collected from multiple perspectives. To facilitate comparability of data with other countries, the study methodology is closely modeled after existing high-quality studies. This report presents the methodology established in the ZULIDAD study.

**Table 1:** Main characteristics of the four published prospective cohort studies on institutional end-of-life care in dementia

Study	Sample	Data sources	Main outcome measures	Main study aims
CareAD [25–27]	*N* = 123 (91 cases of death), census in 3 NH, main inclusion criteria: life expectancy of 6 month or less, dementia diagnosis, receiving or meeting criteria for hospice or palliative care	Chart review (BL, 3 M, PM); surrogate decision-makers (BL, 3 M, PM); physicians (BL); nurses (BL, incomplete information); direct assessment of residents (BL, 3 M)	Medical status (by charts); treatment decisions (by surrogates); quality of life (by caregivers and surrogates); frequency of contact with staff (by surrogates); spiritual and religious beliefs (by surrogates)	Description of health problems, examination of decisions of surrogate decision-makers regarding treatment
CASCADE [5, 16]	*N* = 323 (177 cases of death), main inclusion criteria: CPS 5 or 6, dementia diagnosis, GDS = 7	Chart review, nurses and clinical examination (BL, 3 M, PM2, PM7); REL (BL, PM2, PM7)	EOLD-SM (by nurses); EOLD-CAD (by nurses); EOLD-SWC (by REL); QUALID (by nurses); DSI (by REL)	Description of disease trajectories, resident comfort, clinical decision-making, family satisfaction with care, complicated grief among REL
DEOLD [17, 28]	*N* = 372 (218 cases of death), main inclusion criteria: CPS 5 or 6, GDS = 7	Physician (BL, 6 M, PM), REL (BL, 6 M, PM)	EOLD Scales, PAINAD, QUALID (by physicians and REL)	Description of comfort, symptom burden, pain and family satisfaction with care
EoLO-PSODEC [29, 30]	*N* = 315 (NH), *N* = 181 (home care) (100 cases of death), main inclusion criteria: FAST ≥ =7, life expectancy of more than two weeks	Chart review (bi-weekly), nurses (bi-weekly), physicians (incomplete information)	Diagnosis, ongoing treatment, current prescriptions, appropriateness of prescription (by charts), DS-DAT (by nurses)	Description of treatment and prescription, discomfort, critical decisions

Note. *Abbreviations*: *NH* nursing home, *REL* relatives, *BL* baseline, *3 M* three-monthly, *6 M* biannually, *PM* post mortem, *PM2* post mortem after two weeks, *PM7* post-mortem after 7 weeks, *CPS* cognitive performance score [15, 31], *GDS* global deterioration scale [32], *EOLD-SM/ -SWC/ -CAD* end-of-life in dementia - symptom management/ -satisfaction with care/-comfort at dying [21], *QUALID* quality of life in late-stage dementia scale [24], *DSI* decision satisfaction inventory [33], *PAINAD* pain assessment in advanced dementia [34], *FAST* functional assessment staging [35], *DS-DAT* discomfort scale for dementia of the alzheimer’s type [36]

### Study aims

The aims of the ZULIDAD study are:

1. To describe a sample of Swiss RAD during their last phase of life and thus explore the situation in Switzerland (e.g., How are RAD cared for during the last phase of their life? What are common symptoms and how are they managed?).
2. To compare the perspectives of REL and PN (e.g., What is the level of suffering perceived by REL and PN? How is the quality of life estimated by REL and PN?).
3. To describe disease and care trajectories (e.g., How does care change over time? How do symptoms change over time?).
4. To compare the results with those from other studies/countries (e.g., How is the quality of life of RAD in Switzerland compared to other countries such as the Netherlands?).

## Methods

### Study design

**Fig. 1:**
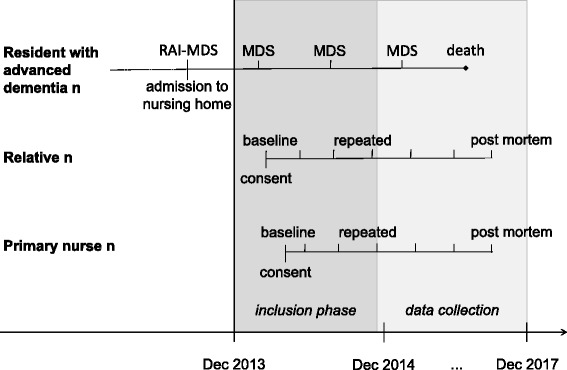
Timeline of data collection in the ZULIDAD study

The ZULIDAD study employs a prospective multi-perspective design. Residents of eleven nursing homes (NH) in the greater Zurich area in Switzerland are followed for three years or until their death. Observational data is collected three-monthly through extensive questionnaires for REL, and PN (see Fig. 1). In addition, routine data from the Resident Assessment Instrument – Minimal Data Set (RAI-MDS), Version 2.0 [15], is collected annually (full assessment) and biannually (abbreviated assessment). In order to facilitate international comparability, the study design, inclusion criteria and applied measurements refer to previous studies, namely CASCADE from U.S. [16], DEOLD from the Netherlands [17] and Dying Well from Belgium [18]. ZULIDAD is a collaborative study with partners from several university centers and departments, nursing homes and the municipal physician service of Zurich. The conduct of the ZULIDAD study was approved by the Ethics Committee of the Canton of Zurich (KEK-ZH-Nr. 2013-0385) and was registered in FORSbase (Ref No 11530), a Swiss online platform for social science studies.

### The ZULIDAD round table

The entire ZULIDAD research process is accompanied by the Round Table (RT) in terms of a participatory research approach. The RT is composed of representatives of three relevant stakeholder groups (REL of RAD; professionals in dementia care, nursing care and palliative care; researchers). Scientific and strategic decisions as well as study results are discussed at the RT on a regular basis. The RT supervises the ZULIDAD study by supporting and advising the research team (e.g., with regard to the selection of variables, the wording of questions or the appropriate interpretation of results). The RT furthermore carries out an independent but related project aiming at the dissemination of the ZULIDAD study results.

### Study setting

The ZULIDAD study is a multicenter study. It is being conducted in eleven NH (ten municipal NH of the City of Zurich, one privately managed NH specialized in dementia care, Sonnweid AG). The municipal NH have a predefined staff ratio, are obliged to have an appropriate skill and grade mix and are certified on a regular basis. Altogether the municipal NH encompass 1’625 beds (ranging from 42 to 334). Based on RAI-MDS data 69 % of the residents had a dementia diagnosis in 2013 (ZULIDAD unpublished data). The privately run NH Sonnweid offers 154 beds which are exclusively for people with dementia.

### Study population

The ZULIDAD study population comprises three groups of subjects: 1) RAD, 2) REL, and 3) PN. Eligibility criteria are presented in Table 2. More than 1’700 RAD were screened, 410 of them met the inclusion criteria and 126 REL and PN gave their informed consent (as of June 2016). Thus, the sample size currently encompasses 126 RAD, REL and PN (3 × 126). The exact sample size will be confirmed in publications after data collection has finished.

**Table 2:** Eligibility criteria for nursing home residents with advanced dementia, relatives and primary nurses

	Inclusion criteria	Exclusion criteria
RAD	- At least one complete RAI-MDS assessment in database - Dementia diagnosis (RAI item I1q (Alzheimer’s Dementia) or I1u (other dementia) - CPS of 5 or 6 - Informed consents by the authorized representatives, following the presumed will of the resident	- Sub-acute or short-term rehabilitative unit - Cognitive impairment due to a major stroke, traumatic brain injury, tumor, or chronic psychiatric condition - Cognitive impairment due to coma
REL	- Informed consent - Proficiency in German	
PN	- Informed consent - Proficiency in German	

Note. *CPS* cognitive performance score, it is composed of five variables from the RAI-MDS [15], scores range from 0-6. Scores of 4-6 identify residents who are severely impaired in their daily decision-making. A CPS score of 5 is comparable to a Mini Mental State Examination score of 5 [37]

*Abbreviations*: *RAD* resident with advanced dementia, *RAI-MDS* resident assessment instrument – minimum data set, *REL* relative, *PN* primary nurse

### Recruitment and informed consent

Recruitment is conducted consecutively in the eleven participating NH. Eligible RAD are identified based on screening the NH’s RAI-MDS databases on a reference date. Subsequently, legally authorized representatives (REL and assistances) and, if the authorized representative cannot be the informant an additional REL, are contacted by postal mail which includes an information sheet describing the study. If they declare interest by returning a prepaid return form, they receive a phone call by a trained research assistant who provides more information about the study and a personal meeting is scheduled if interest persists. The first face-to-face contact with REL includes a detailed clarification of the study procedure, provision of written informed consent (for informant and RAD according to the presumed will) and – if both consents are provided – the completion of the baseline questionnaire. A research assistant then contacts the PN of the RAD directly by telephone (a general information about the study has been provided by the manager of the NH in advance), informs about the study procedure and arranges – if interest is expressed – a personal meeting in order to provide full study information, obtain informed consent and to complete the baseline questionnaire. If REL does not respond to the initial enquiry, they receive one postal reminder.

### Data collection

After the baseline assessment, questionnaires are sent three-monthly and are completed autonomously by the REL and PN (see Fig. 1). If problems occur, participants are requested to contact the research team. Should a RAD decease, the last questionnaire (post mortem) is sent out two weeks (PN) and six weeks (REL), respectively, after death. The duration of the initial face-to-face meeting is 90-120 min, and the repeated and post mortem questionnaires require 45-60 min to complete. If REL or PN fail to submit two questionnaires consecutively, they are excluded from the study. However, unless REL withdraws consent, the RAD remains in the study population, as long as either REL or PN continues to submit the questionnaires. Data collection started in November 2013 and will presumably end in December 2017.

### Instruments

As shown in Table 3, the questionnaires for REL and PN address several topics suggested by the ZULIDAD RT members to play an important role in the last phase of life of RAD. Corresponding variables and instruments were selected based on clinical and research expertise of the investigators and existing studies (indicated in Table 3). Whenever possible, reliable and validated instruments were used, which were – if necessary – translated into German (indicated in Table 3) following ISPOR guidelines [19] and/or slightly adapted to the field of dementia. With few exceptions, questions have a closed-ended response format. Overall, there are two sets of ZULIDAD questionnaires, one for REL and one for PN, which in turn contain three different compositions of variables and instruments – baseline, three-monthly and post mortem. Questionnaires were revised and piloted by the members of the ZULIDAD RT. Data is entered into a central study database which runs on the RedCAP platform [20]. Questionnaire data is complemented by the routinely collected RAI-MDS data [15].

**Table 3:** Data collection elements in the ZULIDAD study

Topic	Instruments	Source	Time
RAD characteristics			
Demographics		REL	BL
Dementia		REL/RAI-MDS	BL
Health status		REL/PN/RAI-MDS	BL/6M
Quality of life	QUALID^abcd^, single item	REL/PN	BL/3M/PM
Pain	BISAD, single item	PN	BL/3M/PM
Suffering	MSSE^a^, single item	PN	BL/3M/PM
Behavioral problems	NPI-Q	PN	BL/3M/PM
Survival time		REL/PN	BL/3M
Care			
Treatment strategy		PN	BL/3M/PM
Current treatments		PN	BL/3M/PM
Symptom Management	EOLD-SM^abc^	REL/PN	BL/3M/PM
Satisfaction with care	EOLD-SWC^abc^, single item, open question	REL	BL/3M/PM
Communication		REL/PN	BL/3M/PM
Trust in staff		REL	BL/3M/PM
Decisions	DSI^ab^	REL	3M/PM
Dying			
Circumstances of dying		REL/PN	PM
Quality of dying	EOLD-CAD^abcd^, QOD-LTC^a^, QODD, FPCS^a^, single item	REL/PN	PM
Advanced planning issues			
Advanced directives		REL/PN	BL/PM
Presumed preferences	PADD^a^	REL	BL
Care agreements		PN	BL/3M/PM
REL characteristics			
Demographics		REL	BL
Wellbeing	WHO-5	REL	BL/3M/PM
Relation to RAD		REL	BL/3M
Knowledge		REL	BL
Attitudes		REL	BL/3M/PM
PN characteristics			
Demographics		PN	BL
Wellbeing	WHO-5	PN	BL/3M/PM
Work		PN	BL/3M/PM
Attitudes		PN	BL/3M/PM

Note. RAD Demographics = Sex, year of birth, religious affiliation, marital status. Dementia = Dementia type, Cognitive Performance Scale. Health status = Overall health status, changes in overall health status, RAI-MDS. Survival time = Estimated survival time, has dying phase started? Treatment strategy = palliative vs. curative. Current treatments = Medical treatments, safety-related measures, psychosocial interventions. Communication = Frequency of and satisfaction with staff/REL communication. Trust in staff = Trust in nursing staff. Decisions = Satisfaction with decisions (DSI), discussions about interventions and whether they lead to decisions. Circumstances of Dying = Cause of death, place of death, attendees, subjective estimation of global dying quality. Quality of dying = *REL:* EOLD-CAD, QOD-LTC, FPCS; *PN:* EOLD-CAD. Advanced directives = Availability, content, were directives adhered to? Care agreements = Regarding medical treatments and safety-related measures. Demographics REL = Sex, year of birth, nationality, religious affiliation, education. Wellbeing REL = Health status, wellbeing (WHO-5), quality of life, emotional burden, feelings of guilt. Relation to RAD = relationship quality before dementia and after dementia, frequency and duration of visits. Knowledge = Knowledge of dementia, palliative care and new legislation. Attitudes = Attitudes towards means of ending or prolonging life. Demographics PN = Sex, year of birth. Wellbeing PN = Health status, wellbeing (WHO-5), quality of life. Work = Work satisfaction, work load, work experiences, qualification

*Abbreviations*: *RAD* resident with advanced dementia, *RAI-MDS* resident assessment instrument – minimum data set, *REL* relative, *BL* baseline questionnaire, *3 M* three-monthly questionnaire, *6 M* six-monthly RAI-MDS, *PM* post mortem questionnaire, *QUALID* quality of life in late-stage dementia scale [24], *BISAD* Observational instrument to assess pain in dementia [23], *MSSE* mini suffering state examination [22], *EOLD-SM/ -SWC/ -CAD* end-of-life care in dementia – symptom management/ -satisfaction with care/ -comfort assessment in dying [21], *QOD-LTC* quality of dying in long-term care [38], *PADD* preferences about dying and death [39], *QODD* quality of dying and death (corresponds with PADD) [40], *FPCS* family perception of care scale [41], *DSI* decision satisfaction inventory [33], *WHO-5* the WHO-five well-being index [42]

^a^newly translated into German, ^b^applied in CASCADE study, ^c^applied in DEOLD study, ^d^applied in the “Dying Well” study

### Statistical analysis

**Fig. 2:**
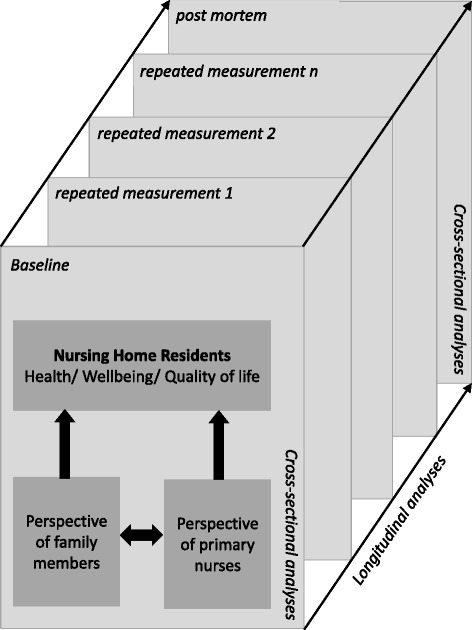
Illustration of proposed data analyses

A first focus will be put on the cross-sectional analysis of baseline and post-mortem data, regarding quality of life, quality of dying and comfort/discomfort and how the perceptions of these measures differ between REL and PN (see Fig. 2). A second focus will be put on longitudinal analyses in order to understand dynamics of change and to identify potential factors that influence stability and deterioration over time. Therefore standard longitudinal data analysis approaches based on the general linear model and multilevel and structural equation models such as latent growth curve or growth mixture models will be applied. In line with previous publications established scales such as EOLD [21], MSSE [22], BISAD [23] or QUALID [24] will be used as primary outcome measures to estimate quality of dying. A third focus will be put on the comparisons with data from other countries, e.g., the Netherlands.

## Discussion

The importance of dementia as a life-limiting condition is increasing. As a consequence, providing end-of-life care in dementia will become one of the most challenging tasks for health care systems. Yet, existing knowledge about palliative care in dementia is scarce. With a prospective cohort design the ZULIDAD study examines the last phase of life of RAD. It is one of the few existing prospective studies on the last phase of life in dementia and it is the first prospective study to describe the situation in the greater Zurich area, Switzerland. The combination of data from REL, PN and RAI-MDS allows a comprehensive description and an approximation to the actual quality of life and quality of dying of the RAD. Furthermore, the multi-perspective approach will help to close knowledge gaps regarding differences in REL’s and PN’s perceptions of central outcome measures at the end of life in dementia. Systematic differences in proxy estimations (e.g., REL and PN) can provide a basis for interventions and interventional studies aiming at optimizing palliative care.

The alignment of the ZULDAD study design, inclusion criteria and applied measures to related studies allows for comparisons between European countries and between Europe and U.S. However, due to the geographical limitation to the greater Zurich area and the limitation to municipal NH and NH specialized in dementia care the study results will neither be generalizable to the French- and Italian-speaking parts of Switzerland nor to all nursing homes in Zurich. The right-censoring of some of the data (because not all RAD will be followed until death) is another bias that needs to be considered when interpreting the data. A unique characteristic of the ZULIDAD study is that it is performed as participatory research project with stakeholder involvement throughout the entire study process. The ZULIDAD RT promotes the practical relevance and the effective knowledge transfer of study results.

In conclusion, the ZULIDAD study provides prospective multi-perspective data on the last phase of life of RAD. This is an important pre-requisite to improve palliative care in advanced dementia in Switzerland and internationally.

## Abbreviations

NH: Nursing home
PN: Primary nurse
RAD: Nursing home resident with advanced dementia
RAI-MDS: Resident assessment instrument – minimum data set
REL: Relatives
RT: Round table
ZULIDAD: Zurich Life and Death with Advanced Dementia study
